# Zur Visualisierung von „Unaussprechlichem“: Geschlechtskrankheiten auf der „Großen Ausstellung für Gesundheitspflege, soziale Fürsorge und Leibesübungen“ (GeSoLei) 1926

**DOI:** 10.1007/s00120-025-02633-2

**Published:** 2025-06-20

**Authors:** Matthis Krischel, Sarah Czirr, Friedrich H. Moll

**Affiliations:** 1 Institut für Geschichte, Theorie und Ethik der Medizin, Centre for Health and Society, Heinrich-Heine-Universität Düsseldorf, Düsseldorf, Deutschland; 2 Institut für Kunstgeschichte, Heinrich-Heine-Universität Düsseldorf, Düsseldorf, Deutschland; 3 Curator, Museum, Bibliothek und Archiv zur Geschichte der Urologie, Düsseldorf – Berlin, Deutschland; 4 Kliniken der Stadt Köln gGmbH, Urologische Klinik, Neufelder Straße 32, 51067 Köln, Deutschland

**Keywords:** Medizingeschichte, Gesundheitsausstellung, Gesundheitskommunikation, Düsseldorf, Öffentliche Gesundheit, History of medicine, Heath exhibition, Health communication, Duesseldorf, Public health

## Abstract

Die GeSoLei (Große Ausstellung für Gesundheitspflege, soziale Fürsorge und Leibesübungen) in Düsseldorf im Jahr 1926 war nicht nur eine der größten Ausstellungen der Weimarer Republik. Im Zentrum stand die oft visuelle Vermittlung von Gesundheitsthemen – darunter auch die Präsentation von Geschlechtskrankheiten. Diese Themen wurden im Spannungsfeld von medizinischer Aufklärung, moralischer Normierung und ästhetischer Inszenierung vermittelt. Die Abteilung „Volkskrankheiten, Volksgebrechen, Volksunsitten“ (Hauptabteilung Soziale Fürsorge) vermittelte Informationen über Tuberkulose, Alkoholismus und Syphiliserkrankungen, die nicht nur medizinisch, sondern auch sozial und moralisch aufgeladen waren. Anhand von Schautafeln, Moulagen und interaktiven Exponaten wurde der Zusammenhang von Sexualität, Krankheit und gesellschaftlicher Verantwortung betont. Die visuelle Strategie folgte dabei einem pädagogischen und oft abschreckenden Prinzip, das durch standardisierte Darstellungsformen eine massentaugliche Ansprache ermöglichte. Die Ausstellung integrierte neben dem Leitkonzept der Sozialhygiene auch Rassen- und Erbgesundheitslehre und rückte Geschlechtskrankheiten in den Kontext der „Volksgesundheit“ und „rationalen Menschenwirtschaft“. Aspekte sexueller Vielfalt oder der Sexualwissenschaft, wie sie etwa von Magnus Hirschfeld vertreten wurden, blieben hingegen ausgespart. Dieser Beitrag analysiert die visuelle und konzeptionelle Ausstellung von Geschlechtskrankheiten auf der GeSoLei als exemplarisches Beispiel für die Medikalisierung und Moralisierung von Sexualität im frühen 20. Jahrhundert – eingebettet in ein komplexes Gefüge aus Wissenschaft, Gesellschaft, Politik und Ästhetik.

## Abstract (en)

The GeSoLei (Great Exhibition for Health Care, Social Welfare, and Physical Exercise) held in Düsseldorf in 1926 was one of the largest exhibitions of the Weimar Republic. At its core was the often visual communication of health-related topics—including the presentation of sexually transmitted diseases. These subjects were conveyed within the complex interplay between medical education, moral normalization, and esthetic staging. The section “Common Diseases, Common Ailments, Common Vices” (Main Section Social Welfare) brought together conditions such as tuberculosis, alcoholism, and syphilis—sicknesses that were not only medically, but also socially and morally charged. Through the use of information boards, wax models (moulages), and interactive exhibits, connections between sexuality, disease, and social responsibility were emphasized. The visual strategy followed a pedagogical and often deterrent approach, which, through standardized forms of representation, enabled communication suited for a mass audience. In addition to the guiding principle of social hygiene, the exhibition also incorporated ideas of racial and hereditary health, placing sexually transmitted diseases within the context of “public health” and “rational population management.” However, aspects of sexual diversity or sexology—as advocated by figures like Magnus Hirschfeld—were deliberately excluded. This article analyzes the visual and conceptual presentation of sexually transmitted diseases at GeSoLei as a representative example of the medicalization and moralization of sexuality in the early 20th century—embedded in a complex web of science, society, politics, and aesthetics.

## Einleitung

Ausstellungen^1^ gehörten in der ersten Hälfte des 20. Jahrhunderts zu den wichtigsten Instrumenten der sog. hygienischen Volksbelehrung. Ihre Aufgabe bestand darin, durch die Vermittlung medizinisch-naturwissenschaftlicher Kenntnisse das gesundheitsrelevante Verhalten der Bevölkerung zu lenken und damit die Entstehung sowie Ausbreitung von Krankheiten zu verhindern. Sie waren damit mehr als der Ausdruck zunehmender Wissenschaftspopularisierung:

Ausstellungen waren auch Foren, auf denen Grenzen menschlicher „Normalität“ ausgelotet wurden [1, 2], sich Aufklärung und Disziplinierung miteinander verbanden und so einschließende und ausgrenzende Effekte gleichermaßen erreichen ließen [3]. Vor diesem Hintergrund war und ist nicht nur die Darstellung von Geschlechtskrankheiten immer durch die jeweilig herrschenden zeitgenössischen Wissenschafts‑, Moral- aber auch damit verbundenen ästhetischen Vorstellungen bestimmt [4].

**Abb. 1:**
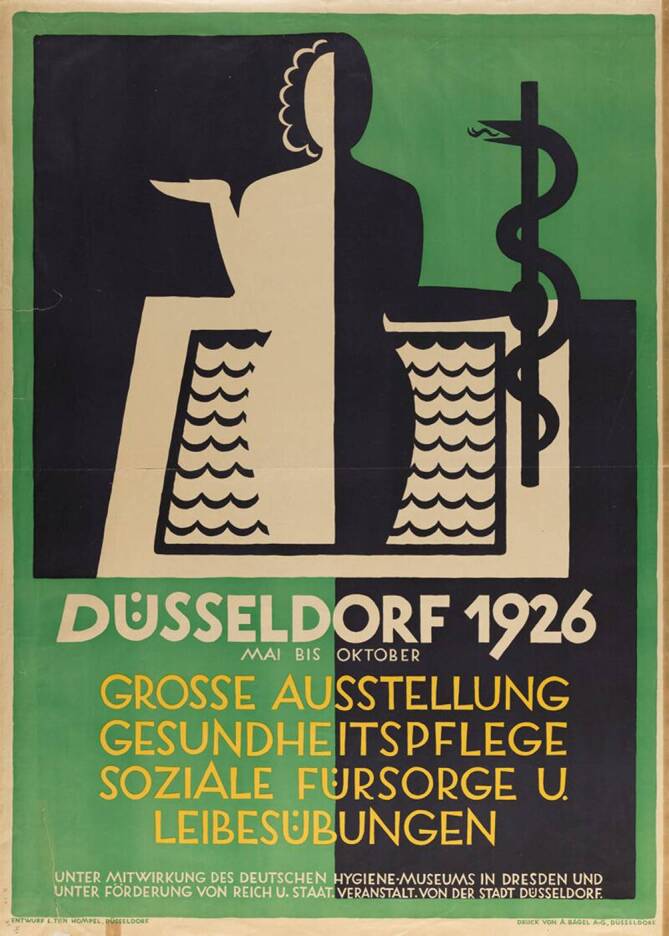
Plakat zur GeSoLei (Große Ausstellung für Gesundheitspflege, soziale Fürsorge und Leibesübungen), Wellcome Images

All diese Aspekt kommen auch bei der Großen Ausstellung für Gesundheitspflege, soziale Fürsorge und Leibesübungen zum Tragen: Im Jahr 1926 gehörte die die GeSoLei (Abb. 1), das Akronym wurde bereits von den Zeitgenossen heftig diskutiert^2^, als *„eine Angelegenheit des deutschen Volkes*“ zu den besonderen Projekten der Stadt Düsseldorf nach dem verlorenen ersten Weltkrieg und der *„Schmach von Versailles“* [5]. Neben den drei Themenbereichen Gesundheitspflege, soziale Fürsorge und Leibesübungen fügten sich auch Industrie- und Kunstausstellungen sowie Publikumsattraktionen etwa in Form eines Wellenbades problemlos ein. Die Ausstellung war eine Schau dessen, was Mitte der 1920er-Jahre schlechthin als modern angesehen wurde. In ihren widersprüchlichen und ambivalenten Darstellungen der Errungenschaften, Krisen und Zukunftsvisionen der 1920er-Jahre spiegelt sie anschaulich das gesellschaftliche und kulturelle Klima der Zeit wider.

**Abb. 2:**
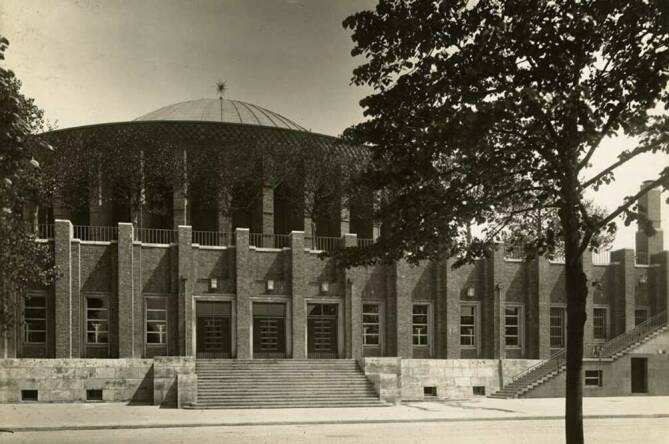
Planetarium auf der GeSoLei (Große Ausstellung für Gesundheitspflege, soziale Fürsorge und Leibesübungen), heute Tonhalle, eine Konzerthalle in Düsseldorf. (Stadtarchiv Düsseldorf, mit freundl. Genehmigung)

Die Hauptinitiatoren waren der Industrielle Ernst Poensgen (1871–1949), Vereinigte Stahlwerke, der Leiter der Kinderklinik der Düsseldorfer Medizinischen Akademie, Arthur Schloßmann (1867–1932) sowie der Oberbürgermeister und spätere Innenminister der BRD nach dem Zweiten Weltkrieg, Robert Lehr (1883–1956). Der Direktor der Kunstgewerbeschule Düsseldorf und Professor an der Kunstakademie Düsseldorf, Wilhelm Kreis (1873–1955), zeichnete für die Dauerbauten, wie auch das spätere Haus des Hygienemuseums in Dresden, verantwortlich. (Abb. 2). Es sollte das größte, im thematisch-sachlichen Bereich am breitesten aufgestellte und im Aufklärungs- und Identifikationsanspruch ambitionierteste Ausstellungsprojekt der jungen Weimarer Republik werden. Im Mittelpunkt der Ausstellung [6–9], unter Schirmherrschaft des 1925 gewählten Reichspräsidenten Paul von Hindenburg (1847–1934) und des österreichischen Bundeskanzlers Michael Hainisch (1848–1940), die nach Eröffnung durch Reichskanzler Hans Luther (1879–1962)^3^ und des letzten demokratischen Landeshauptmanns der preußischen Rheinprovinz Hans Horion (1876–1933) am 8. Mai 1926 von mehr als 7,5 Mio. Besuchern gesehen wurde^4^ und ein Areal von ca. 400.000 m^2^ einnahm (die größte während der Weimarer Republik), sollte die gesamte Hygiene stehen. Denn nur durch sie, verbunden mit Sport, sollte nach Aussagen führender Wissenschaftler der Zeit das deutsche Volk genesen. Die soziale Hygiene, zu der auch die Sexualhygiene gehörte wie auch der Sport, der ebenfalls als ein wichtiger Gegensatz zu einer ungezügelten Sexualität aufgefasst wurde, waren wesentliche Hauptanliegen. Hierdurch sollte eine „rationelle Menschenwirtschaft“ [10] ermöglicht werden, durch welche die Wirtschaft wieder in Schwung kommen sollte. Die politische und soziale Zielsetzung der Ausstellung war die Erziehung zum gesundheitsbewussten und leistungsfähigen Menschen.

**Abb. 3:**
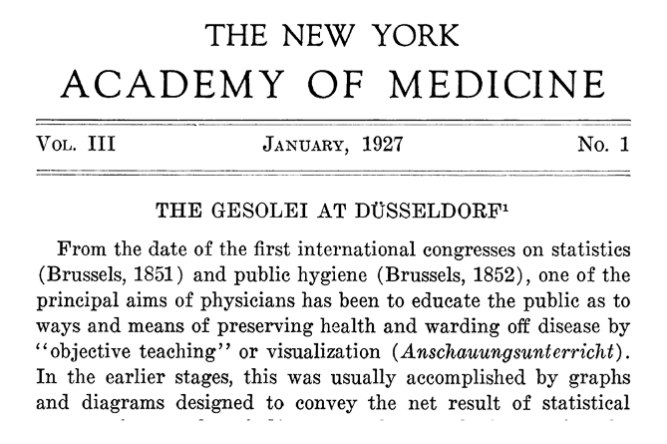
Ausriss „The GeSoLei at Düsseldorf“ Fielding Hudson Garrison (1870–1925) Medizinhistoriker mit Mitherausgeber des Index Medicus Bull N Y Acad Med. 1927, Jan;3(1) 1–6. Dies zeigt gut die internationale Breitenwirkung dieser größten „Gesundheitsschau“ während der Weimarer Republik. (Repro Moll-Keyn, mit freundl. Genehmigung)

Bis heute ist die GeSoLei Teil der städtischen Erinnerungskultur und die Ausstellung war bereits Gegenstand historischer und kunsthistorischer Studien [11–16] und populärer Beiträge [17, 18]. Die hier vorgestellte Untersuchung geht der Frage nach, wie Geschlechtskrankheiten mit Blick auf das Gesamtkonzept dieser größten „Gesundheitsmesse“ der Weimarer Republik mit einem Millionenpublikum und internationaler Rezeption, ähnlich wie bei einer Weltausstellung, unter ausstellungsstrategischen, sozialhygienischen und gesundheitspolitischen Aspekten visuell vermittelt wurden (Abb. 3 und 4).

**Abb. 4:**
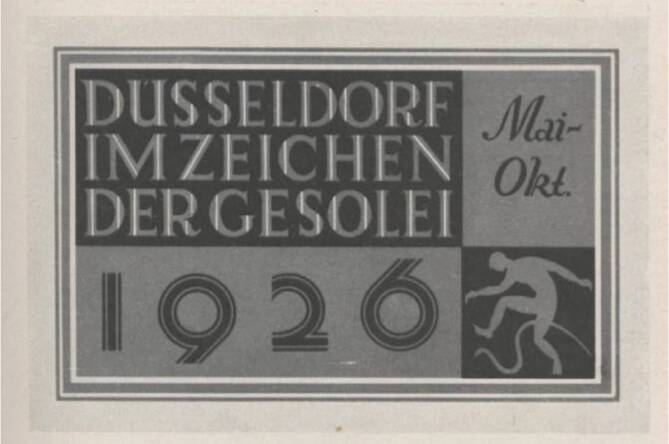
Werbemarke der GeSoLei (Große Ausstellung für Gesundheitspflege, soziale Fürsorge und Leibesübungen) vom 8. Mai bis 15. Oktober 1926: Richard Schwarzkopf Gebrauchsgraphik Rhein Ruhr 1926 Heft 3, S. 27 online: Slub https://www.arthistoricum.net/werkansicht/dlf/132201/63/0/. (Repro Moll-Keyn, mit freundl. Genehmigung)

## Geschlechtskrankheiten und Sexualhygiene auf der GeSoLei

Gesundheitsausstellungen sind paradigmatische Formate für die Popularisierung sozialhygienischer Wissensbestände, besonders in der Zeit vom wilhelminischen Kaiserreich bis zu dem Beginn des Zweiten Weltkriegs. Aus zeitgenössischer Sicht wird ihre Funktion folgendermaßen beschrieben:

„Sie bringen in gemeinverständlicher Form den jeweiligen Stand der Gesundheitswissenschaft und ihrer Anwendung im Leben des Einzelnen und des Volkes möglichst lückenlos zur Schau, gewahren historische Rückblicke und kennzeichnen so die Fortschritte. Ihre Mannigfaltigkeit bürgt dafür, daß jeder Besucher seine Rechnung findet, zum Nachdenken bewogen wird und viel lernen kann – ganz zu schweigen von den tiefen Anregungen und dem fruchtbaren Erfahrungsaustausch für hygienische Fachleute, für Künstler, Architekten, Techniker. Sie müssen demnach von Zeit zu Zeit geschaffen werden.“ [19]

Für die Präsentation des Themenkomplexes der Geschlechtskrankheiten und Sexualhygiene, die unter dem Oberbegriff „Volkskrankheiten, Volksgebrechen, Volksunsitten“ (Hauptabteilung Soziale Fürsorge, Halle 30) neben Tuberkulose, Krebs und Alkoholismus arrangiert wurde, zeichneten neben dem Deutschen Hygienemuseum in Dresden und der Deutschen Gesellschaft zur Bekämpfung der Geschlechtskrankheiten mit Hermann Roeschmann (1873–1950; Geschäftsführer; [20])^5^ auch Carl Stern (1864–1935) als lokaler Repräsentant in seiner Eigenschaft als Leiter der Hautklinik der Medizinischen Akademie Düsseldorf verantwortlich.^6^

Geschlechtskrankheiten wurden auf der GeSoLei vorrangig dem Prinzip der Prävention untergeordnet und waren u. a. Teil der Ausstellung in einer Beratungsstelle der Landesversicherungsanstalt. Die Einflechtung an dieser Stelle zeigt, dass der Gesunderhaltung des „Volkskörpers“ ein wichtiger Beitrag bei der Seuchenbekämpfung beigemessen wurde, der hilft, dem Einzelnen Lasten zu tragen: „Fürsorge durch Versicherung“ [21]. Im Ausstellungskatalog heißt es dazu:

„Die Bestrebungen, den Geschlechtskrankheiten durch vorbeugende Maßnahmen zu geben, sind in der Abteilung ‚Geschlechtskrankheiten‘ stark in den Vordergrund gerückt worden. In Tafeln und Bildern, die größtenteils dem Deutschen Hygiene-Museum, aber auch dem Ausland entstammen, wird Lehrern und Eltern Material an die Hand gegeben, um die Jugendlichen in das heikle Gebiet des Geschlechtslebens einzuführen. Plakate aus verschiedenen Ländern zeigen, wie die Öffentlichkeit auf die Gefahren, welche in den Geschlechtskrankheiten liegen, hingewiesen wird. Die Krankheitserscheinungen sind in Wachs oder farbigen Bildtafeln naturgetreu wiedergegeben und bilden für alle, welche sich belehren lassen wollen, eine ernste Warnung.“^7^

Somit knüpften die Ausstellungsmacher der GeSoLei an die erste Hygieneausstellung in Dresden 1911 und das museologisch-technische Wissen des Hygienemuseums dort an und ließen auch Erfahrungen, welche die bereits 1902 gegründete Deutsche Gesellschaft zur Bekämpfung der Geschlechtskrankheiten auf Wanderausstellungen gewonnen hatte, einfließen.

Die Ausstellungsmacher gingen von der Prämisse aus, dass durch die Popularisierung von medizinisch-naturwissenschaftlichem Wissen das gesundheitsrelevante Verhalten breiter Bevölkerungskreise verbessert werden konnte [22]. Marta Fraenkel (1896–1976), eine Hauptakteurin der Ausstellungsumsetzung, wies darauf hin, dass die Präsentation eines Themas in allen drei Bereichen der Ausstellung durchaus von den Ausstellungsmachern gewünscht war, um neben klinisch-medizinischen, seucheninfektiologischen oder mikrobiologischen Gesichtspunkten auch die sozialhygienischen Aspekte oder Behandlungsansätze durch Sport hervorzuheben [23].

**Abb. 5:**
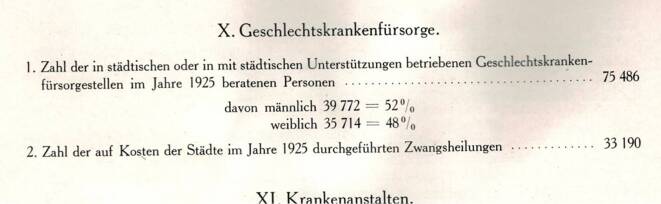
„Geschlechtskrankenfürsorge“ aus: Schloßmann A 1927 Bd II, S. 820. (Repro Moll-Keyn, mit freundl. Genehmigung)

**Abb. 6:**
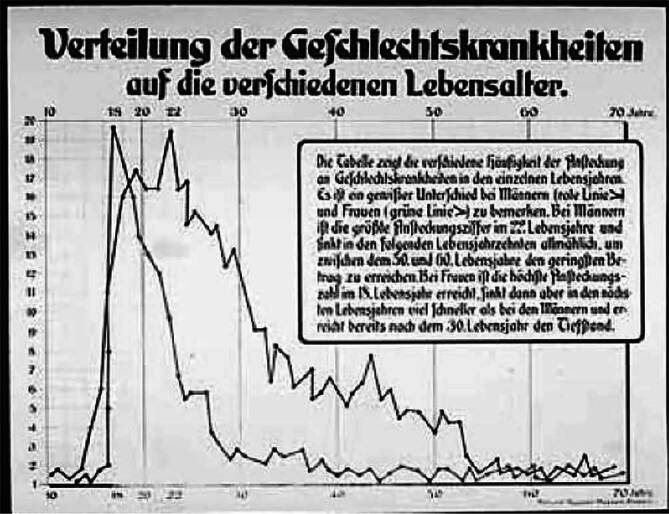
Schautafel des Hygienemuseums Dresden zur Verteilung der Geschlechtskrankheiten in verschiedenen Altersstufen. DHGM DHMD 1999/984

**Abb. 7:**
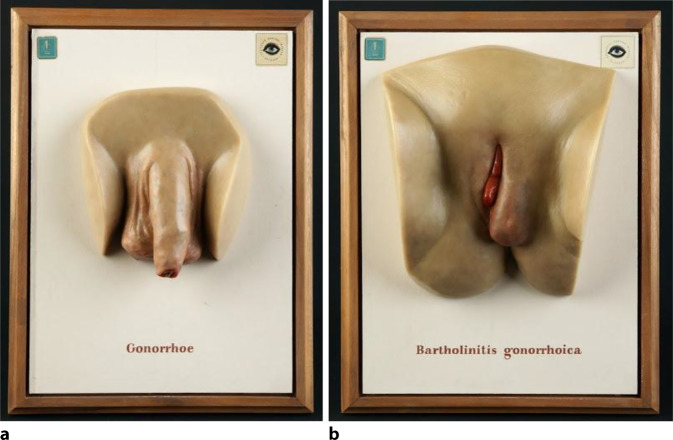
**a**,**b** Die Moulagen aus den primären Abformungen kurz nach 1900 wurden durch das Hygienemuseum Dresden bis 1989 als Lehrmaterialien vertrieben. Hier typische Schaukästen mit Marke des Hygienemuseums Dresden, 1960er-Jahre. Solche Exponate waren auch auf der GeSoLei (Große Ausstellung für Gesundheitspflege, soziale Fürsorge und Leibesübungen) ausgestellt. Moulagen waren als Visualisierungsobjekte seit Ende des 19. Jahrhunderts gut verfügbar. Abbildungen Museum, Bibliothek und Archiv, Deutsche Gesellschaft für Urologie. (Repro Moll-Keyn, mit freundl. Genehmigung)

**Abb. 8:**
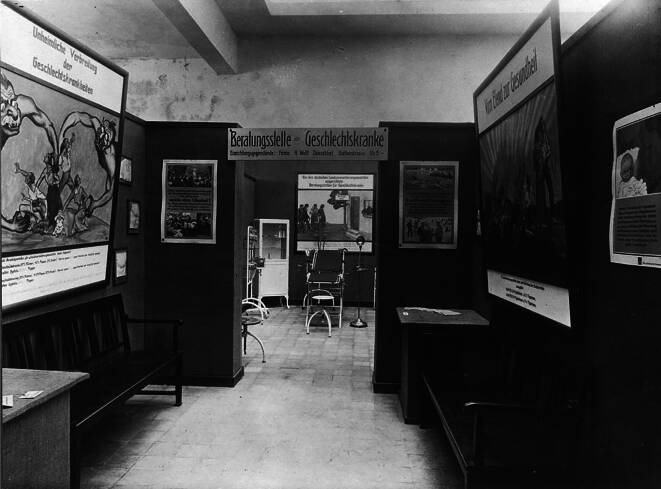
„Beratungsstelle für Geschlechtskranke“ der Landesversicherungsanstalt DHMD 2013 830 24. (Mit freundl. Genehmigung)

**Abb. 9:**
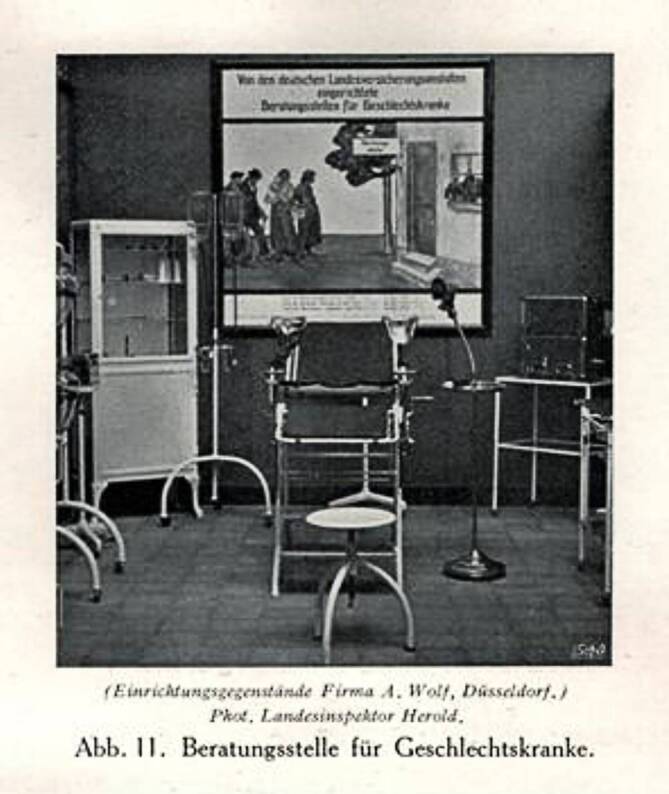
Detailansicht des hinteren Raumteiles „Beratungsstelle für Geschlechtskranke“ der Landesversicherungsanstalt aus Schloßmann 1927, Bd II, S. 794. (Repro Moll-Keyn, mit freundl. Genehmigung)

Aus den Abbildungen und den Sammlungsbeständen des Hygienemuseums in Dresden lassen sich für die Visualisierung der Geschlechtskrankheiten Tafeln und Objekte zum größten Teil rekonstruieren. Bei der Präsentation wurde das in der Visualisierung häufig verwendete Prinzip der Abschreckung genutzt, wie der Text der Schautafeln in der vorderen Ausstellungzone, aber auch Statistiken zu den Geschlechtskrankheiten, markant veranschaulichen (Abb. 5, 6, 7, 8, 9, 10 und 11).

**Abb. 10:**
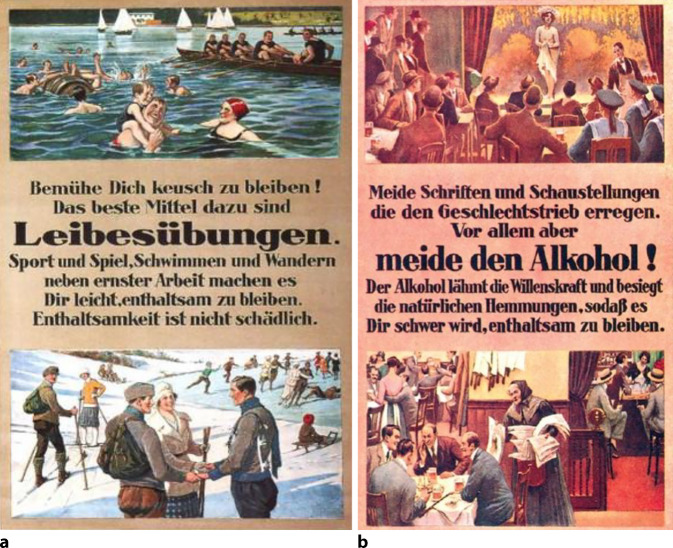
**a**,**b** Infotafeln auf der Kopfseite des Vorraums „Bemühe Dich keusch zu bleiben“ sowie „Meide Schriften und Schaustellungen“ DHMD 1995 26 Lehrtafeln. (Mit freundl. Genehmigung)

**Abb. 11:**
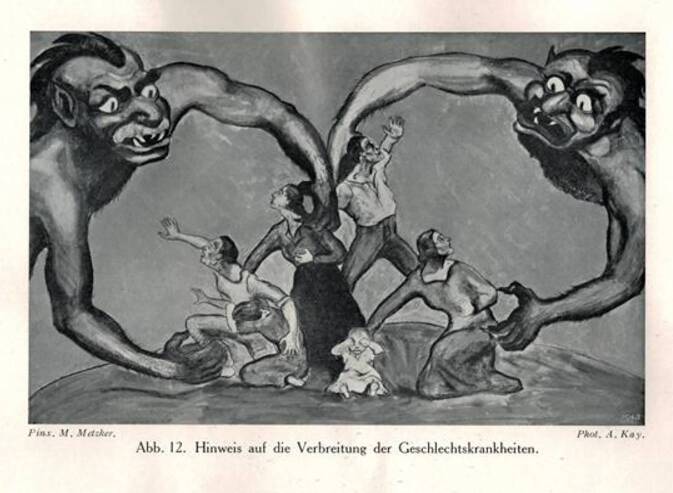
Abbildung links an der Wand des Vorraums aus Schloßmann 1927 Bd II S 795 Schloßmann. (Repro Moll-Keyn, mit freundl. Genehmigung)

Die Moulagen, die das Hygienemuseum in Dresden lieferte, wurden an Zahl etwas zurückgenommen zugunsten großflächiger Darstellungen mit eindrücklicher Wirkung (Abb. 6a,b). Auf die Idee, Moulagen neben der primären medizinische Befunddokumentation auch für die Gesundheitsaufklärung breiter Kreise und für den Kampf gegen die Geschlechtskrankheiten zu nutzen, kamen der Dresdener Philanthrop August Linger (1861–1916, „Erfinder“ des Mundwassers Odol® und Initiator der ersten Hygieneausstellung in Dresden; [24, 25]) sowie Eugen Galewski (1864–1935, Deutsche Gesellschaft zur Bekämpfung der Geschlechtskrankheiten; [25, 26]).

**Abb. 12:**
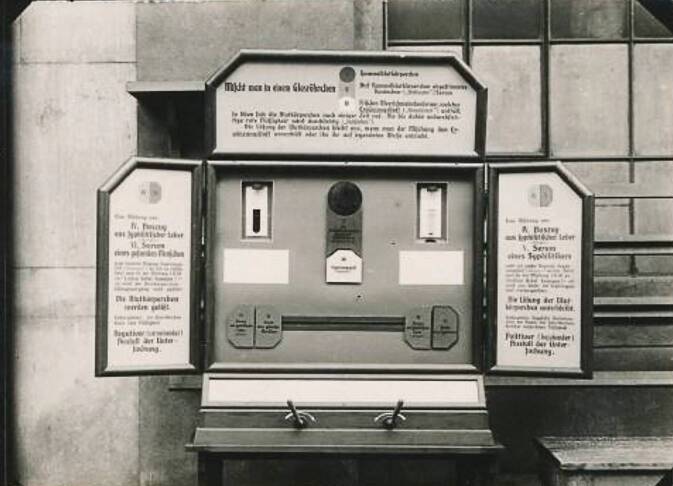
Wassermann Reaktion Beleuchteter interaktiver Schaukasten, DHMD 2015 457. (Mit freundl. Genehmigung)

Die unterschiedlichsten Ausstellungsbereiche wurden über eine modern wirkende Präsentation^8^ unter didaktischen Aspekten zusammengestellt, um so möglichst ein Massenpublikum zu erreichen. Aus Kreisen der Düsseldorfer Kunstakademie bereitgestellte Werkstätten sorgten „mit einer fast standardisierten künstlerischen Visualisierungsstrategie für eine einheitliche Erscheinung und eine auf gezielte Besucheransprache abgestimmte Vermittlung“ [27]. Didaktisches Ausstellungsmaterial – dazu zählen Lehrtafeln, aber auch Schau- und bewegliche Demonstrationsobjekte – nehmen ab der Wende zum 20. Jahrhundert zu.^9^ So werden auf der GeSoLei neben den 414 Dioramen als zeitgemäße Medien der Immersion auch interaktiv beleuchtete Schaukästen wie bei der Wassermann-Reaktion zur Diagnostik der Lues als leibhaftige Erlebnisformen ausgestellt (Abb. 12).

Diese Formen der unmittelbaren Ansprache der Besucher wurden auch bei anderen Ausstellungsbereichen – die Industrie bot beispielsweise Einblicke in Fertigungsprozesse durch den Aufbau von Maschinen und Produktionsstraßen^10^ – angewendet.

## Geschlechtskrankheiten und Rassenhygiene

**Abb. 13:**
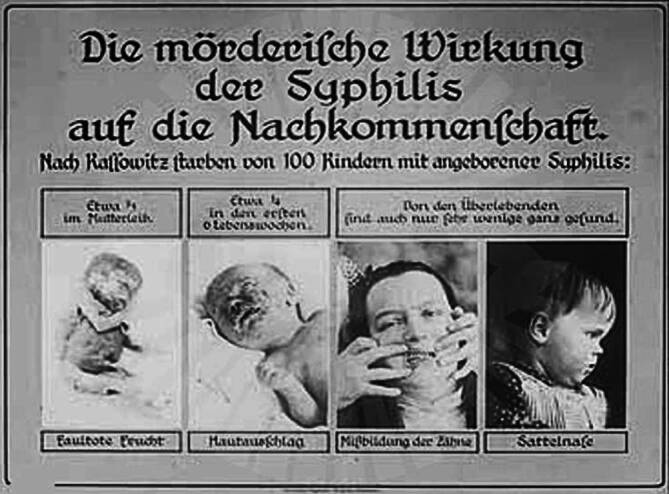
Die mörderische Wirkung der Syphilis auf die Nachkommenschaft (nach Kassowitz) DHMD DHMD 1999/2174 Bild 67/„Geisteskrankheiten und abnormes Seelenleben/Lichtbildreihe 59“ (70 LB). (Mit freundl. Genehmigung)

Die „II. Hauptgruppe: Erblichkeitslehre und Rassenhygiene (Eugenik)“ (Ge II, Halle 25, 1 Stock) war ausschließlich dem Themenkomplex unter Mitarbeit der führenden Wissenschaftler der Weimarer Republik wie Erwin Bauer (1875–1933) oder Fritz Lenz (1887–1976), die ein weit rezipiertes Lehrbuch der Zeit publiziert hatten, gewidmet^11^ [28]. Das Thema war, wie auch in der Dauerausstellung des Hygienemuseums in Dresden, auch in anderen Bereich der GeSoLei präsent und wirkmächtig (Abb. 13). Heiko Zielke bemerkt dazu:

„Während ein Museum für Rassenkunde [1934] eine alte aber unerfüllt gebliebene Forderung rassistischer Interessengruppen darstellte, gab es bereits in der Weimarer Zeit und auch davor eugenische Ausstellungen. So zeigte die GeSoLei 1926 unter tatkräftiger Mitwirkung des Deutschen Hygienemuseums Dresden eine Gruppe ‚Erblichkeitslehre und Rassenhygiene‘, die allerdings trotz einzelner populärer Darstellungen weitgehend auf einen Expertendiskurs zugeschnitten war.“ [29]

„In der Hauptabteilung Gesundheitspflege wurden sozial- und rassenhygienische Aspekte nebeneinandergestellt und so ideologische Schnittmengen demonstriert [30]. Eine Koje beschäftigte sich in der Abteilung ‚Der Mensch in seinen gesundheitlichen Beziehungen zu Pflanzen und Tieren‘ mit der Vererbung in der Biologie.“ [31]

Der Leiter des Hygienemuseums in Dresden Martin Vogel (1887–1947) erläutert die Zusammenhänge darüber hinaus wie folgt:

„Über die Keimschädigungen durch Blei, Alkohol, Nikotin und Geschlechtskrankheiten führte der Gedankenweg weiter zu den Fragen der praktischen Rassenhygiene und Bevölkerungspolitik. Die Frage der Ehetauglichkeitszeugnisse […] hatte darin ebenso Platz wie die einer vernünftigen Erziehung zum Verständnis von Ehe und Fortpflanzung, die Gefahr der geringen Kinderzahl in den sozial höherstehenden Schichten ebenso wie die gefährlichen Verschiebungen, die durch den Krieg in der Zusammensetzung der Bevölkerung in den am Krieg beteiligten Staaten eingetreten sind.“ [32]

Der „Eheberatung“ wurde damit ein wichtiger Platz eingeräumt:

„In der Untergruppe ‚Eheberatung‘ wird zunächst die Notwendigkeit dargetan, daß die Ehebewerber auf die leibliche und geistige Gesundheit ihrer Partner ihr Augenmerk richten sowie auf deren gesunde Abkommenschaft. Insbesondre werden die unheilvollen Folgen, die die Volksseuchen Tuberkulose, Geschlechtskrankheiten und Alkoholismus für die Nachkommenschaft mit sich bringen, dem Beschauer klargelegt.“^12^

Von der Adoption ausgeschlossen werden sollten Kinder, deren Eltern an Geschlechtskrankheiten oder Tuberkulose litten oder deren Mutter als „leichtfertig“, also sexuell zügellos, galt. Somit galten körperliche und moralische Kriterien als vererbbar [33]. Zur Visualisierung war die Aufarbeitung dieser Zusammenhänge durch eindrucksvolle Statistiken ein besonders geeignetes Mittel, um beim Publikum keine nennenswerten Zweifel an den vermeintlichen Fakten aufkommen lassen [34, 35].

## Leerstellen

Während die Prävention und Behandlung von Geschlechtskrankheiten als Teil des Spektrums der sozialen Fürsorge einen wichtigen Teil der GeSoLei darstellte, blieben andere, in der Weimarer Republik durchaus schon diskutierte Aspekte der Sexualwissenschaft außen vor [36]. Hierzu gehörten neben der Hormonforschung [37] auch die breiten Bereiche der Homosexualität und Transgeschlechtlichkeit, die zur gleichen Zeit von Magnus Hirschfeld (1868–1935) an seinem Berliner Institut für Sexualwissenschaften sowohl erforscht als auch in praktische Patientenversorgung und auch Ausstellungen umgesetzt wurden [38, 39]^13^. Diese Leerstellen zeigen auch die Grenzen des Anspruchs der GeSoLei auf, die öffentliche Gesundheit der Weimarer Republik zu verbessern. Es scheinen eben nicht alle Teile der Bevölkerung hierbei mitgedacht worden zu sein.

## Schlussfolgerung – Zusammenfassung

Die Gesundheit und körperliche Fitness des Einzelnen und die öffentliche Gesundheit sowie die Fokussierung auf einen neuen Leistungsbegriff waren zentrale Aspekte der Düsseldorfer Ausstellung GeSoLei, die auch fast 100 Jahre später noch immer aktuell sind.

Den Geschlechtskrankheiten als wichtiger Seuche mit besonderer gesellschaftlicher Auswirkung für die „Volksgesundheit“ wurde von den Ausstellungsmachern entsprechend den Erfahrungen der Deutschen Gesellschaft zur Bekämpfung von Geschlechtskrankheiten und der Hygieneausstellung in Dresden ein besonderer Raum zur Präsentation eingeräumt. In der fachübergreifenden Perspektive durch die Verbindung von Kunst, Wissenschaft und Wirtschaft als mikrokosmischer Verdichtung zentraler Diskurse der Weimarer Republik lassen sich durch die Analyse der GeSoLei Rückschlüsse über Körperbilder sowie die Visualisierungsstrategien der hygienischen Volksbelehrung dieser Zeit ziehen.

## Footnotes

1 Die Forschung zu Ausstellungen ist vielfältig, wie nur zwei hier aufgeführte Titel belegen mögen: Geppert, A C. T. 2002 Welttheater: Die Geschichte des europäischen Ausstellungswesens im 19 und 20. Jahrhundert – Ein Forschungsbericht Neue Pol Lit 1 42–44; Weinert S 2017 Der Blick in den Körper Gesundheitsausstellungen vom späteren Kaiserreich bis in die Weimarer Republik. Ordnungssysteme. Studien zur Ideengeschichte der Neuzeit 50. De Gruyter Oldenbourg Berlin. Im aktuellen gesellschaftlichen (Stichwort Humboldt Forum) und wissenschaftlichen Diskursen (Ausdruck hiervon ist die Etablierung von Studiengängen wie Curatorial Studies) wird die Rolle von Ausstellungen einer kritischen Neubewertung unterzogen. Für ein Forschungsfeld wie es die Ausstellung GeSoLei darstellt, ist es deshalb unerlässlich diese neuen Forschungsansätze mit einer systematischen Quellenauswertung zu verknüpfen.

2 Kozella L 1926 Brief von der Gesolei Salzburger Chronik 16. Juli 1926 S 2–3, Online-Zugriff über https://anno.onb.ac.at/. Zugegriffen 12.01.2025.

3 Nach Wechsel zur Regierung des Reichskanzlers Wilhelm Marx (1863–1946),der aus Köln gebürtig war, besuchte dieser am 12. Juli 1926 ebenfalls die Gesolei.

4 Die Weltausstellung in Barcelona im Jahr 1929 hatte 5,8 Mio. Besucher.

5 Hermann Roeschmann war bei der Ausarbeitung des Gesetzes zur Bekämpfung der Geschlechtskrankheiten 1927 publizistisch vielfach hervorgetreten vgl Borelli S Vogt HJ, Kreis M 1992 Geschichte der deutschen Gesellschaft zur Bekämpfung der Geschlechtskrankheiten. Blackwell, Berlin S 46–67.

6 Amtlicher Katalog Grosse Ausstellung fuer Gesundheitspflege Soziale Fürsorge Leibesübungen Düsseldorf 1926. Bagel Druck, Düsseldorf S. 165.

7 Amtlicher Katalog Grosse Ausstellung fuer Gesundheitspflege Soziale Fürsorge Leibesübungen Düsseldorf 1926. Bagel Druck, Düsseldorf S. 173.

8 Heiko Zielke stellt zum Düsseldorfer Reichs- und Landesmuseums für Wirtschaft heraus: „Mit Dioramen, Schautafeln, Modellen und mechanischen Apparaten blieb das Museum auch in der Nachkriegszeit weitgehend den Gestaltungsformen der Gesolei verpflichtet.“ Vgl. Zielke, Heiko, Die große Masse des Volkes wirtschaftlich denken lehren. Zur Geschichte des Düsseldorfer Reichs- und Landesmuseums für Wirtschaft 1926 bis 1958, in: Jg. 15, Heft 1 (2000) S. 65–94, hier S. 92.

9 Stercken A (2002) Die Gesolei als Schaubild des Körpers. Sektionen, Überblick, in: Hans Körner, Angela Stercken (Hrsg.): Kunst Sport und Körper. GeSoLei. 1926–2002, Bd. 1, Ostfildern-Ruit, S. 99–123, hier S. 101. In der bisherigen Forschung fehlt eine systematische Erfassung und Analyse – und diese nicht nur in Hinblick auf ihre Funktion, sondern auch auf ihre Gestaltung – der auf der GeSoLei vertretenen Ausstellungsmaterialien. Methodische Ansätze aus den Bildwissenschaften, dem New Materialism und ANT sind hierfür unerlässlich. Alexander C. T. Geppert stellt in diesem Zusammenhang fest, dass Museum- und Ausstellunggeschichte „in einer neuen Form von Netzwerkanalyse gegenseitige Einflussnahmen und wechselseitige Interdependenzen einzelner Ausstellungsinstanzen exakt nachzuzeichnen“ seien. Vgl. Osten, P 2005 Hygieneausstellungen: Zwischen Volksbelehrung und Vergnügungspark, Dt Ärztebl 102: A 3085–3088.

10 Thomas Großbölting widmet sich in seiner Studie ausführlich der Ausstellung von Arbeit und Industrie und beschreibt Ausstellungen als „Deutungsangebote und Erfahrungsorte für den technischen und industriellen Fortschritt, die Ausbildung der Konsumgesellschaft, die Verlockungen und Angebote einer entstehenden Freizeitindustrie.“ Großbölting, Thomas, „Im Reich der Arbeit“. Die Repräsentation gesellschaftlicher Ordnung in den deutschen Industrie- und Gewerbeausstellungen 1790–1914, Berlin/Boston 2008, S. 11. Sarah Czirr hat in ihrer Untersuchung erarbeitet, wie auch über das Medium Skulptur der zeitgenössische Begriff von Arbeit entworfen oder verfestigt wurde und bezieht dabei auch die aus dem Bereich der Medizin erwachsenen Körperdiskurse mit ein. Vgl. Sarah Czirr, „Arbeitende Bilder“. Die Skulptur im Deutschen Kaiserreich zwischen künstlerischer Aneignung und sozialer Wirklichkeit, Bielefeld 2018.

11 Amtlicher Katalog Grosse Ausstellung fuer Gesundheitspflege Soziale Fürsorge Leibesübungen Düsseldorf 1926. Bagel Druck, Düsseldorf, S. 85–86.

12 Amtlicher Katalog Grosse Ausstellung fuer Gesundheitspflege Soziale Fürsorge Leibesübungen Düsseldorf 1926. Bagel Druck, Düsseldorf S 169.

13 Für Wien vergl. Mildenberger F (2025) im Druck Urologie und Sexualforschung in Wien in der ersten Hälfte des 20. Jahrhunderts in: Moll, F., Halling, T, Shariat S Urologie in Österreich Von Wien in die Welt, Springer, Heidelberg ISBN 978-3-662-70887‑3.

## References

[1] Weinert S (2017) Der Blick in den Körper Gesundheitsausstellungen vom späteren Kaiserreich bis in die Weimarer Republik. Ordnungssysteme. Studien zur Ideengeschichte der Neuzeit 50. De Gruyter Oldenbourg, Berlin, S 221 10.1515/9783110469011

[2] Weinert S (2015) Prävention durch Aufklärung. Gesundheitsausstellungen in der ersten Hälfte des 20. Jahrhunderts. Gesundheitswesen. 10.1055/s-0035-1563024

[3] Labisch A (1992) Homo Hygienicus. Gesundheit und Medizin in der Neuzeit. Campus, Frankfurt, S 111

[4] Weinert S (2017) Der Blick in den Körper Gesundheitsausstellungen vom späteren Kaiserreich bis in die Weimarer Republik. Ordnungssysteme. Studien zur Ideengeschichte der Neuzeit 50. De Gruyter Brill, Berlin 10.1515/9783110469011

[5] Teich-Balgheim O (1926) Grundgedanken, allgemeine Bedeutung und äußere Gestaltung der Gesolei. Adler, Düsseldorf, S 5

[6] Garrison FH (1927) The Gesolei at Düsseldorf. Bull N Y Acad Med 3(1):1–6 19311556 PMC2393578

[7] Price GM (1926) Industrial hygiene abroad and the hygienic exhibition at Düsseldorf. Am J Public Health 16(12):1202–1204. 10.2105/ajph.16.12.1202 10.2105/ajph.16.12.1202 PMC1321492 18012022

[8] NN (1926) Düsseldorf Health Exhibition Brit Med. J 1 (3413) 951–956, insb955

[9] Grünewald M (1926) Die „Ge-So-Lei“ in Düsseldorf. Jpn Deutsch Z Wiss Tech 4(6):159–161

[10] Poensgen E (1927) Die wirtschaftliche Bedeutung der Gesolei. In: Schloßmann A (Hrsg) GeSoLei Grosse Ausstellung Düsseldorf 1926 für Gesundheitspflege Soziale Fürsorge und Leibesübungen Bd I, Schwann, Düsseldorf, S 15–17 (hier S. 16)

[11] Stöckel S (1991) Die große Ausstellung über Gesundheitspflege, Sozialfürsorge und Leibesübungen – Gesolei – 1926 in Düsseldorf. In: Dt. Ges Gesch Med Nat Technik (Hrsg) Ideologie der Objekte – Objekte der Ideologie. Naturwissenschaft, Medizin und Technik in Museen des 20. Jahrhunderts 73. Jahrestagung, Mannheim, 2.–5. Oktober 1990 Deutsche Gesellschaft für Geschichte der Medizin, Naturwissenschaft und Technik e. V., Wenderoth Kassel, S 31–38 (90. Gründungsjubiläum 1991)

[12] Körner H, Genge G, Stercken A (Hrsg) (2004) Kunst, Sport und Körper. Bd 1. 1926–2002. Eine Ausstellung über die Ausstellung GeSoLei. Hatje Cantz, Ostfildern-Ruit

[13] Wiener J (Hrsg) (2001) Die GeSoLei und die Düsseldorfer Architektur der 20er-Jahre. J. P. Bachem, Köln

[14] https://www.geschichte.hhu.de/abteilungen/ausgelaufene-abteilungen/europaeische-expansion-1/unsere-forschung/duesseldorfer-globalgeschichte-lehrforschung/die-gesolei-in-duesseldorf/die-gesolei-gebaeude. Zugegriffen: 12. Dez. 2020

[15] Körner H, Genge G, Stercken A (Hrsg) (2004) Kunst, Sport und Körper. Bd 2. 1926–2004. Methoden und Perspektiven. VDG, Weimar

[16] Körner H, Genge G, Stercken A (Hrsg) (2004) Kunst, Sport und Körper. Bd 3. 1926–2004. Bilder einer Ausstellung. VDG, Weimar

[17] (2014) 15. Oktober 1926 Die GeSoLei endet in Düsseldorf. https://www.br.de/radio/bayern2/sendungen/kalenderblatt/1510-gesolei-duesseldorf-neues-koerperideal-tonhalle-100.html. Zugegriffen: 12. Dez. 2024

[18] Wir in den wilden Zwanzigern online: 2/2. https://programm.ard.de/TV/wdrfernsehen/wir-in-den-wilden-zwanzigern--2-2-/eid_281112226618698. Zugegriffen: 21. Dez. 2024

[19] Frey G (1927) Gedanken über hygienische Volksbelehrung, ihre Wege und Hilfsmittel. In: Frey G (Hrsg) Gedanken über hygienische Volksbelehrung, ihre Wege und Hilfsmittel. Springer, Berlin, S 3–38 10.1007/978-3-662-31586-6_1

[20] Roeschmann H (1926) Der Gesetzentwurf zur Bekämpfung der Geschlechtskrankheiten nach der zweiten Lesung im bevölkerungspolitischen Ausschuß. Dtsch Med Wochenschr 52(34):1434–1436. 10.1055/s-0028-1127625

[21] Appelius, Schellmann (1927) Fürsorge durch Versicherung. In: Schlossmann A (Hrsg) Ge So Lei Grosse Ausstellung Düsseldorf 1926 für Gesundheitspflege Soziale Fürsorge und Leibesübungen, Bd. 2. Schwann, Düsseldorf, S 783–822 (insb. S. 820)

[22] Weinert S (2015) Der „Tod“ als Argument Strategien der hygienischen Volksbelehrung vom späten Kaiserreich bis zum Anfang der 1960er-Jahre. A Soz Gesch 55:107–131

[23] Fraenke M (1926) Organisatorisches und Methodisches auf der Gesolei. In: Amtlicher Katalog Grosse Ausstellung für Gesundheitspflege Soziale Fürsorge Leibesübungen Düsseldorf 1926. Bagel, Düsseldorf, S 41–48

[24] Funke U (1993) Karl August Lingner – Leben und Werk eines sächsischen Großindustriellen. Med. Fak., Dresden, S 48 (Dissertation)

[25] Osten PH (2005) Hygieneausstellungen zwischen Volksbelehrungen und Vergnügungspark. Dtsch Ärztebl A102:3085–3088

[26] Moll F, Görgen A, Fangerau H (2013) Urologische Moulagen Vergessene dreidimensionale Dokumente zwischen Universitätssammlung und Panoptikum – eine sterbende Präsentationsform auch der urologischen Museologie. Urologe 52:1118–1127. 10.1007/s00120-013-3275-5 10.1007/s00120-013-3275-5 23933709

[27] Stercken A (2002) Die Gesolei als Schaubild des Körpers. Sektionen, Überblick. In: Körner H, Stercken A (Hrsg) Kunst Sport und Körper. GeSoLei. 1926–2002, Bd. 1. Ostfildern-Ruit, S 99–123 (hier S. 108)

[28] Fangerau H (2003) Der „Baur-Fischer-Lenz“ in der Buchkritik 1921–1940: Eine quantifizierende Untersuchung zur zeitgenössischen Rezeption rassenhygienischer Theorie. Med Hist J 28:57–81. 10.2307/25805337 14509235

[29] Zielke H (2000) Die große Masse des Volkes wirtschaftlich denken lehren. Zur Geschichte des Düsseldorfer Reichs- und Landesmuseums für Wirtschaft 1926 bis 1958. Gesch W 15:65–94 (S. 83)

[30] Fehlemann S, Woelk W (2002) Der „Wiedergesundungsprozess des deutschen Menschen“. Zum Verhältnis von Gesundheit, Hygiene und Gesellschaft auf der Düsseldorfer Gesolei. In: Kunst, Sport Körper| GE SO LEI 1926–2002 Eine Ausstellung über die Ausstellung GeSoLei. VDG, Weimar, S 186–192 (insb. S. 188)

[31] Siemon H (1927) Der Mensch in seinen gesundheitlichen Beziehungen zu Pflanzen und Tieren. In: Schloßmann A (Hrsg) Ge So Lei Grosse Ausstellung Düsseldorf 1926 für Gesundheitspflege Soziale Fürsorge und Leibesübungen, Bd. 2. Schwann, Düsseldorf, S 540–570

[32] Vogel M (1927) Das Deutsche Hygiene-Museum auf der Gesolei. In: Schloßmann A (Hrsg) GE-SO-LEI, Bd. 2. Schwann, Düsseldorf, S 449–474 (insb. 466)

[33] Stöckel S (1991) Die große Ausstellung über Gesundheitspflege, Sozialfürsorge und Leibesübungen – Gesolei – 1926 in Düsseldorf. In: Dt. Ges Gesch Med Nat Technik (Hrsg) Ideologie der Objekte – Objekte der Ideologie. Naturwissenschaft, Medizin und Technik in Museen des 20. Jahrhunderts Vorträge von der 73. Jahrestagung, Mannheim, 2.–5. Oktober 1990 Deutsche Gesellschaft für Geschichte der Medizin, Naturwissenschaft und Technik e. V., Wenderoth Kassel, S 31–38 (90. Gründungsjubiläum 1991)

[34] Nikolow S (2002) Die graphisch-statistische Darstellung der Bevölkerung Bevölkerungskonzepte in der Gesundheitsaufklärung in Deutschland vor 1933. In: Mackensen R (Hrsg) Bevölkerungslehre und Bevölkerungspolitik vor 1933. Leske + Budrich, VS, Opladen Wiesbaden, S 297–214 10.1007/978-3-322-92254-0_16

[35] Nikolow S (2005) Statistische Bilder der Bevölkerung in den großen Hygieneausstellungen als Wissensobjekte. In: Reulecke J, Mackensen R (Hrsg) Das Konstrukt Bevölkerung vor im und nach dem „Dritten Reich“. VS, Wiesbaden, S 476–488 10.1007/978-3-322-80803-5_20

[36] Krischel M, Kühl R, Mahr D (2023) Neue Forschungsfragen in der Geschichte der deutschsprachigen Sexualwissenschaft und Sexualmedizin. Urologie 62(11):1204–1210 37428184 10.1007/s00120-023-02091-8 PMC10630189

[37] Hansson N, Krischel M, Södersten P, Moll F, Fangerau H (2020) “He gave us the cornerstone of Sexual Medicine”—A Nobel plan but no Nobel Prize for Eugen Steinach. Urol Int 104:501–509 32172253 10.1159/000506235 PMC7592645

[38] Herrn R (2022) Der Liebe und dem Leid: Das Institut für Sexualwissenschaft 1919–1933. Suhrkamp, Frankfurt

[39] Kühl R (2022) Der Große Krieg der Triebe: Die deutsche Sexualwissenschaft und der Erste Weltkrieg. Die deutsche Sexualwissenschaft und der Erste Weltkrieg. transcript, Bielefeld

